# Gamma radiation-induced enhancement of biocontrol agents for plant disease management

**DOI:** 10.1016/j.crmicr.2024.100308

**Published:** 2024-11-07

**Authors:** Mahsa Rostami, Abozar Ghorbani, Samira Shahbazi

**Affiliations:** Nuclear Agriculture Research School, Nuclear Science and Technology Research Institute (NSTRI), Karaj, Iran

**Keywords:** Biological control, Gamma radiation, Antagonists, Mutagenesis, Plant disease control

## Abstract

Gamma radiation-induced mutations in microorganisms can enhance their properties for the biological control of plant diseases. Mutant strains of *Bacillus subtilis* were found to have improved antifungal properties against *Aspergillus flavus* and increased production of biosurfactants and biofilms. Furthermore, combining gamma radiation with antagonists was more effective in controlling *Penicillium expansum* postharvest than either treatment alone. A major focus of this research was on *Trichoderma* species, which have shown an enhanced ability to control plant diseases through increased production of antifungal metabolites such as hydrolytic enzymes, antibiotics, and total phenols. The mechanism by which gamma radiation alters the genotype of microorganisms is the destruction of double-stranded and single-stranded DNA, resulting in changes in the genome or nucleic acid molecule, altering the antagonistic properties of microorganisms. Sensitivity to radiation is determined by the size of an organism's chromosomes, and the effect on microorganisms is primarily based on DNA or RNA disruption. Molecular analysis of gamma radiation mutants has been used to understand changes in genome composition, including downregulated genes related to secondary metabolism, cytochrome P450 s, carbohydrate-active enzymes, peptidases, and hydrophobins. Gamma radiation thus offers a promising method to induce beneficial genetic changes in microorganisms, enhancing their efficacy in the biological control of plant diseases.

## Highlights

• Gamma radiation improves biocontrol agents for plant disease management.
• Mutant *Bacillus subtilis* strains show enhanced antifungal properties.
• Gamma-irradiated microorganisms increase biosurfactant and biofilm production.
• Enhanced *Trichoderma* species increase antifungal metabolite production.
• Gamma radiation modifies microorganisms' genotype for better biocontrol.

## Graphical abstract

**Figure:**
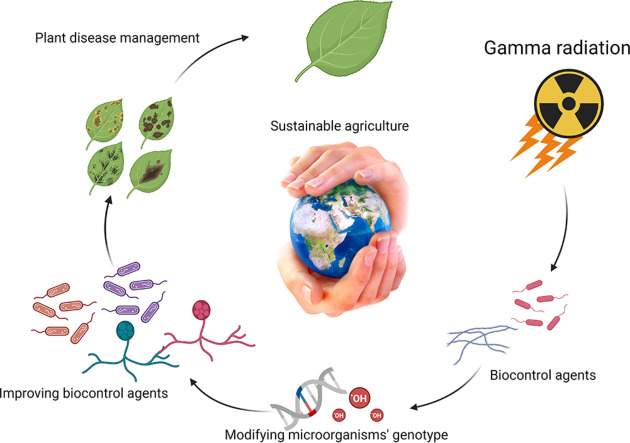

## 1 Introduction

Plant pathogens, including nematodes, fungi, bacteria, mycoplasmas, viruses, and viroids, cause significant losses in crop quality and productivity. Various techniques have been used to prevent and control plant diseases, including chemical fertilizers or insecticides. However, their unfavorable results and excessive use have caused significant damage to the soil environment and increased pollution. Thus, efforts have been made to increase the use of environmentally friendly methods for plant disease control. Biological control through microorganisms—such as antagonistic biocontrol agents (BCAs), in particular, *Pseudomonas* spp., *Bacillus* spp., *Burkholderia* spp., and *Trichoderma* sp.—has gained prominence among these methods (Lahlali et al., 2022). Using microorganisms like bacteria and fungi to combat phytopathogens offers a safer and more sustainable alternative or supplement to chemical pesticides by enhancing plant defense mechanisms and suppressing disease through various modes of action (Zehra et al., 2021). In this approach, the most effective microorganisms are selected and mass-produced in laboratories for agricultural application. This process, known as ‘augmentative biological control’, aims to enhance the natural suppression of plant diseases by increasing the population of these beneficial organisms in the field (Köhl et al., 2019). Various factors, including environmental conditions and large-scale application methods, can influence the effectiveness of these agents.

To date, many beneficial microorganisms have been studied, most of which are fungi and bacteria with unique capabilities to control plant diseases (Raymaekers et al., 2020). These microorganisms have direct or indirect mechanisms of action, including antibiosis, mycoparasitism, induced resistance, growth promotion, and competition for space and nutrients (Thambugala et al., 2020) most important and effective fungi and bacteria against plant pathogens include *Trichoderma, Gliocladium, Aspergillus, Fusarium, Paecilomyces, Agrobacterium, Alcaligenes, Arthrobacter, Bacillus, Enterobacter, Erwinia, Pseudomonas, Rhizobium, Serratia, Stenotrophomonas, Streptomyces*, and *Xanthomonas* (Bonaterra et al., 2022; Huilgol, Nandeesha, and Banu, 2022). In 2017, one hundred microbial control agents were registered in many countries (Köhl et al., 2019). However, despite their proven capability in laboratory settings, the lack of consistent efficiency of available biocontrol agents in field applications remains a significant limiting factor for their broader use (Raymaekers et al., 2020). Factors such as their performance under varying environmental conditions, challenges in large-scale trials, and mass production and formulation issues have reduced their widespread adoption (Bamisile et al., 2021). In addition, biological control agents often face the problem of shelf life, maintaining viability during transportation and storage, and their susceptibility to environmental stresses such as temperature and humidity fluctuations, which limits their practical use in the field (Marrone, 2023). Compounding these challenges, pathogens can develop mechanisms to evade the action of biological control agents, undermining their ability to effectively control diseases. Ultimately, the success of biological control agents depends not only on their inherent properties, but also on a comprehensive understanding of pathogen biology, environmental conditions, and interactions with plants, highlighting the complexity of integrating these agents into sustainable agricultural practices. Addressing these challenges is crucial to fully realize the potential of biological control agents in sustainable agriculture (Lahlali et al., 2022).

Several methods have been used to increase the efficacy of microbial biological control agents, including combining them with other control agents, such as lower fungicide doses, and discovering better microorganisms with improved mechanisms (Palmieri et al., 2022). In this sense, the induction of genetic mutations is one of the fastest and most effective methods for screening microorganisms and selecting strains with antagonistic potential. The results of this method can lead to an increase in antimicrobial metabolites, host colonization, and longer persistence in the micro-ecosystem (Mirmajlessi et al., 2018). This approach accelerates the natural evolutionary process, providing a broader range of biocontrol traits in a shorter timeframe. Gamma radiation is a technique that can induce genetic mutations in microorganisms. Although the process is not entirely predictable, it has successfully led to beneficial mutations in *Trichoderma* (ThM7, TgM1, and TvM17) (at gamma radiation doses under 0.25 kGy), *B. subtilis* BRBac4, *Bacillus siamensis* BRBac21, and *Streptomyces cavourensis* BRAcB10 (0.5–3.0 kGy), resulting in enhanced biocontrol properties (Manikandan et al., 2022; Zaker Tavallaie et al., 2022). This review addresses efforts to use gamma radiation to enhance the antagonistic potential of microorganisms in controlling various plant diseases, including fungal and bacterial pathogens, particularly in economically important crops such as tomatoes, potatoes, and cucurbits. The selection criteria for this narrative review were based on identifying diseases with significant economic impact and crops that are key targets for biological control measures.

## 2 The mechanism of gamma radiation in changing the genotype of microorganisms

**Fig. 1:**
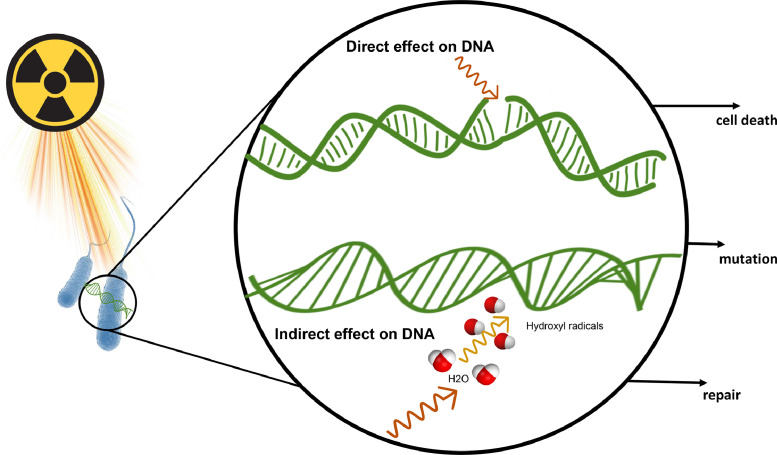
Schematic representation of DNA mutation by gamma radiation, showing the direct and indirect effects of radiation. These cause mutation, repair, or cell death.

Gamma rays are a type of high-energy electromagnetic radiation that are emitted by radioactive isotopes like cobalt-60. These rays can directly affect DNA and cause single-stranded and double-stranded breaks in the DNA molecule, which alters the genome (Jeong and Jeong, 2018). In addition, the absorption of radiation in the treated material leads to an ionization process, which causes stable atoms or molecules to become charged and unstable. This ionization occurs due to the indirect interaction of photons with atoms, causing the ejection of electrons and generating free radicals and excited atoms. One of the most well-known free radicals is hydroxyl, which can combine with nucleic acid molecules. As a result, the effect of irradiated rays on microorganisms is primarily based on the disruption of DNA or RNA, leading to the prevention or destruction of cell proliferation or changes in the antagonistic properties of the microorganisms due to the alteration of the nucleic acid molecule. Free radicals and chemical bonds are responsible for causing changes in microorganisms (Hall and Giaccia, 2006) (Fig. 1).

The sensitivity of microorganisms to radiation is determined by the size of their chromosomes. Organisms with larger chromosomes are more sensitive to radiation. This sensitivity is determined by their ability to repair nucleic acid damage rather than their innate resistance to radiation (Hewitt and Leelawardana, 2014; Mirmajlessi et al., 2018). In this context, 'larger chromosomes' refers to organisms with a more extensive genomic content or a higher DNA content per chromosome, which affects their ability to repair radiation-induced damage, such as DNA repair enzymes (Nikjou, 2003). Moreover, chromosome size is a key cellular factor that determines the radiosensitivity of microorganisms, and circular RNAs (circRNAs), as components derived from the genome, play a crucial role in modulating this sensitivity. CircRNAs play a significant role in various cellular processes, including binding and transporting RNA-binding proteins, generating protein isoforms, and regulating transcription and alternative splicing. They are also crucial in the cellular response to radiation. For instance, circPVT1 has been linked to radiosensitivity, acting as a decoy for microRNAs such as let-7, which influences cellular responses to radiation by regulating proteins that prevent senescence. Similarly, circ-AKT3, another important circRNA, affects the PI3K/AKT/mTOR pathway, a critical regulator of cancer cell radioresistance. Different isoforms of circ-AKT3 can either increase or decrease resistance to radiation, demonstrating the complex role of circRNAs in radiation response. Gamma irradiation has been shown to significantly alter circRNA expression, with some circRNAs being uniquely expressed in irradiated cells, indicating their involvement in post-radiation repair mechanisms. The effects of gamma radiation at varying doses, such as 0.25 Gy and 2.5 Gy, have been studied, showing circRNAs’ dynamic role in modulating cellular repair and survival, highlighting their potential as biomarkers for radiation exposure and response (Reindl et al., 2023). Moreover, the total lipid mass of microorganisms is related to their radiosensitivity, such that an increase in the total lipid content of molds and actinomycetes leads to an increase in radioresistance. This is likely due to the protective properties of lipids, which can shield cellular components from radiation-induced damage (Aziz et al., 1997).

## 3 Appropriate dose of gamma radiation

In addition to chromosome size and the ability to repair nucleic acid damage, the dose of gamma radiation plays a crucial role in determining the radiosensitivity of microorganisms. To improve the quality and efficiency of biocontrol agents, it is crucial to evaluate the specific characteristics of the microorganism, such as its radiosensitivity, its DNA repair capacity and its general metabolic activity. A beneficial feature of radiation exposure would be the ability of a microorganism to maintain its viability and continue to function effectively after treatment, while a detrimental feature would be increased susceptibility to DNA damage or impaired metabolic activity, which could affect its performance (Arena et al., 2014). Selecting the appropriate dose therefore requires careful consideration of these factors as well as the intended purpose of treatment to optimize the desired results. In the case of *Trichoderma* species, a beneficial characteristic after gamma radiation exposure would be the ability to maintain its biocontrol activity, such as producing hydrolytic enzymes that attack plant pathogens. Conversely, a detrimental characteristic would involve excessive DNA damage leading to a reduction in spore germination or a loss of ability to produce these enzymes, ultimately diminishing its effectiveness as a biocontrol agent (Ghasemi et al., 2019).

There are three methods for inactivating microorganisms in food based on irradiation dose: radappertization, radicidation, and radurization. The most powerful method is radappertization, which generally uses a dose between 25 and 45 kGy, resulting in no detectable living microorganisms (except viruses). The radicidation method uses a lower dose, generally between 2 and 8 kGy, which inactivates non-spore-forming bacteria that cannot be detected by bacteriological methods. The lowest dose is used in the radurization method, which is in the range of 0.4–2.5 kGy. The exposure time for each method, whether radappertization, radicidation, or radurization, needs to be optimized based on the specific characteristics of the sample, such as its texture, density, and composition. For instance, dense samples may require longer exposure times to achieve effective microbial inactivation, while more delicate textures may necessitate shorter times to preserve the quality of the product. Therefore, optimizing both the dose and exposure time is crucial to ensure microbial safety without compromising the structural integrity or sensory attributes of the treated food (World Health Organization, 1999; Harder et al., 2017). In plant pathology, gamma irradiation has also proven effective in controlling pathogens such as *Fusarium* and *Botrytis* species that are common in crops. By selecting the appropriate radiation dose, it is possible to inactivate these pathogens without damaging plant tissue or reducing the efficacy of biocontrol agents used in plant disease management (Chu et al., 2015; Sreenivasa et al., 2009).

High-dose gamma radiation can impair genomic DNA integrity in bacteria, with a dose of 54.3 kGy effectively inactivating bacterial spores, vegetative cells, and any resulting microbial colonies formed after exposure (Broomall et al., 2016). However, in the case of *B. subtilis*, mutation (NMBCC50025 isolate) induced by gamma radiation between 0.1 kGy to 1.2 kGy did not result in differences in the 16S rRNA gene compared to the wild type, indicating that the appropriate dose of gamma radiation did not affect the nature of the bacteria (Nie et al., 2018). Actinomycetes, fungi, and invertebrates are eliminated by 10 kGy in most soils, while most soil bacteria are also eliminated by 20 kGy (McNamara et al., 2003).

In another study, the microbial population in soil was irradiated with doses of 1, 4, 16, 32, 64, 256, 512, 1024, and 2048 kg roentgen (kr.) to determine the survival rate of fungi and bacteria. As the dose increased, the survival rate of fungi and bacteria decreased, with the greatest reduction observed at a dose greater than 64 kr (Popenoe and Eno, 1962).

For improving the biocontrol properties of *Lacticaseibacillus paracasei* (ATCC NO.4356, 2 and ATCC NO. 11,981), using 15 doses of gamma irradiation (0–5 kGy) showed that a dose of 3.5 kGy could increase the antibacterial activity of the supernatant (Khalil et al., 2020). Irradiation of *Beauveria bassiana* with gamma doses of 400 and 700 Gy had a significant effect on killing the spiny bollworm (Kandil et al., 2022). In another study, mutation of *Trichoderma aureoviride* (Tv59) was performed with nine different doses (0–450 Gy), with the 250 Gy dose introduced as the optimal dose since it had an inhibitory effect on spore germination (Soufi et al., 2021a).

According to Table 1, microbial population reduction begins at a dose of 1 kGy, and high doses such as 17 and 20 kGy can render the environment microbe-free. The appropriate dose range to increase the efficacy of biocontrol agents varies by species but typically falls between 0.25 and 3.5 kGy. However, depending on the type of microorganism studied, this dose range must be carefully examined and tested to select the optimal dose. It is crucial to note that not all biocontrol agents will respond uniformly to radiation, and the species-specific response should be considered when determining the appropriate dose.

**Table 1:** The effect of different doses of gamma radiation on different materials.

*The dosage unit is kilogray

(World Health Organization, 1999; Harder et al., 2017; Khalil et al., 2020; Soufi et al., 2021; Kandil et al., 2022).

High doses of gamma radiation are commonly used to study the genomic and functional characteristics of microorganisms, such as mutations, gene expression changes, and alterations in metabolic pathways. This approach can be seen as a form of reverse engineering, which has been commonly employed in plant models such as *Arabidopsis* and rice to unravel genetic and metabolic networks. However, there are relatively few references to the use of this method in microorganisms. In this context, Trichoderma has been positioned as a model organism, offering a detailed analysis and description that provides valuable insights into how gamma radiation-induced mutations can alter genomic and metabolic traits. Genomic study of *Trichoderma virens* mutant M7, the mutant induced by 125 k rad (1.25 kGy) gamma radiation and suppressed in mycoparasitism, using RNA-Seq and whole-genome sequencing methods has helped to understand the genes downregulated in this mutant and the changes in the genome composition of the mutant. Biosynthesis of secondary metabolites was downregulated in M7 and 13 out of 73 metabolites were detected. Additionally, 463 genes were downregulated in M7, including those involved in secondary metabolism (such as genes encoding NRPSs, PKSs, terpene cyclases, modifying enzymes, transporters, and transcription factors), cytochrome P450 s, carbohydrate-active enzymes, peptidases, and hydrophobins. Whole-genome sequencing further revealed that the M7 mutant lacked two major secondary metabolism gene clusters (PKS6 and Tex9 clusters), eight transcription factors (two of which are associated with secondary metabolism gene clusters), five carbohydrate metabolism genes, two genes related to cell signaling, and 11 oxidoreductases (Pachauri et al., 2020). These findings suggest that gamma radiation-induced mutations can have a significant impact on the genome composition of microorganisms, affecting their ability to produce secondary metabolites and carry out essential biological processes.

## 4 Disadvantages of using gamma radiation

While gamma radiation offers a multitude of benefits, it is important to acknowledge the potential drawbacks that must be carefully considered when employing this technique. Firstly, the application of high doses of gamma radiation can induce undesirable alterations in the physical and chemical properties of the materials undergoing treatment. Such alterations may impact the texture, flavor, color, and even the nutritional value of food products, potentially leading to reduced consumer acceptance and marketability (Harrell et al., 2018).

In addition, while gamma radiation is an effective method for eliminating pathogens, it is a non-selective process. This implies that both detrimental and beneficial microorganisms are inactivated during treatment, which may result in the disruption of the natural microbial equilibrium. In food products, this can result in a reduction in probiotic bacteria or the promotion of spoilage due to the loss of competitive, non-pathogenic microorganisms. Furthermore, the handling and utilization of gamma radiation give rise to concerns pertaining to safety. The technology in question relies on the use of radioactive isotopes, which require strict regulatory oversight and specialized infrastructure. Such measures include the implementation of protective protocols to ensure the safety of operators, as well as the establishment of protocols to prevent accidental radiation exposure. The mishandling or mismanagement of radioactive materials has the potential to result in significant adverse effects on human health and the environment (Pricaz and Uţă, 2015).

An additional factor to be taken into account is the economic viability of gamma radiation. The establishment and maintenance of gamma irradiation facilities necessitates a substantial initial capital investment, which may prove to be a significant financial obstacle for smaller enterprises. Furthermore, operational costs, including the necessity for continuous monitoring and compliance with safety regulations, contribute to the financial burden, thereby limiting the adoption of this technology by smaller producers (Pahladsingh, 2004).

When considering the use of gamma radiation for enhancing biocontrol agents, analogous constraints must be taken into account. Although gamma radiation can enhance the effectiveness of microbial biocontrol agents by inducing beneficial mutations, it may also damage critical microbial functions or reduce the viability of beneficial organisms. It is therefore essential to optimize radiation doses with the utmost care in order to maximize benefits while minimizing potential risks (Soufi et al., 2021b). Although gamma radiation offers considerable promise in food processing, pathogen control, and the enhancement of biocontrol agents, its deployment must be weighed against its drawbacks to guarantee safe, effective, and economically viable results.

## 5 The effect of gamma rays on the characterization of biocontrol agents

Ionizing radiation such as gamma, X-rays, alpha, or beta is produced by electromagnetic waves or particles emitted by atoms. Gamma rays, which come from the excited nuclei of radioactive elements like cesium-137 and cobalt-60, have a high penetrating power (Shahi et al., 2021). The use of gamma rays has been beneficial in agriculture for years (Feldmann et al., 2019; Piri et al., 2011) and has been found to improve various properties of biological agents, such as enhancing their antimicrobial activity, increasing their resilience to environmental stressors, and promoting their ability to colonize host plants (Mirmajlessi et al., 2018). In the context of biological control, gamma rays have been used to modify the biotic properties of pathogens, parasites, and predators used to control pests and diseases in crops (Table 2).

**Table 2:** Microorganisms that were studies under the effect of gamma-ray for biocontrol of plant diseases.

Microorganisms under the effect of gamma-ray	The effect of gamma-ray	Against	Dose of gamma ray (kGy)	Reference
*Actinomycete* strains: SA2, SA6, SB11, SG4, SG5, SJ9 and K19	increase the ability of antifungal activity	*Fusarium sporotrichiodes, Rhizoctonia solani* and *Sclerotium rolfsii*.	0.4–8	(Rugthaworn et al. 2007)
*Bacillus subtilis* UTB1	Enhancement of biosurfactants and biofilm production	*Aspergillus flavus* R5	0.1- 3	(Afsharmanesh et al. 2013)
*Bacillus thuringiensis* subsp*. Kurstaki, Metarhizium anisopltiae* (Metsch)*, Heterorabditis bacteriophora* (Poinar) BA1*, Steinernema carpocapsae* (Weiser) A11	Change the biological and life parameters	*Spodoptera littoralis* (Boisd.)	0.015, 0.03 and 0.06	(Amer et al. 2015)
*Bacillus thuringiensis* subsp*. Kurstaki, Beauveria bassiana* (Balsamo)	potentiate the effects of biological agents	Pectinophora gossypiella (Saund), Earias insulana (Boisd.), Oxycarenus hyalinipennis (Costa)	0.4 and 0.7	(Amer et al., 2019)
*Bacillus amyloliquefaciens* (HM6)	enhancement of biofilm production	Salt stress	1, 1.5, 2, 2.5, and 3	(Gaafar et al. 2018)
*Lactobacillus acidophilus* ATCC NO.4356*, Lactobacillus rhamnosus* ATCC NO. 11,981	increased the antimicrobial activity	An edible coating on enhancing the storage of tomato	3.5	(Khalil et al., 2020)
*Gliocladium virens*	Different phenotype, growth rate, sporulation and antagonistic potential	*Sclerotium rolfsii* and *Fusarium oxysporum* f.sp. ciceri	1.25	(Mukherjee and Mukhopadhyay 1993)
*Trichoderma atroviride* ATCC 74,058 (P1)	Enhance antagonism and induction of plant systemic disease resistance	*Rhizoctonia solani* 19*, Pythium ultimum* 8*, Botrytis cinerea* 26	not mention	(Brunner et al. 2005)
*Gliocladium virens* (GV1 and GV3)	Potential mycoparasite	Sclerotial plant pathogens	0.5, 0.75, 1, and 1.25	(Jash et al. 2006)
*Gliocladium virens,Gliocladium deliquescens*	Increase the pathogenicity of biological agents against pathogen	*Sclerotium rolfsii*	0, 0.25, 0.5, 1.0, 1.5, 2.0, 2.5, and 3.0	(El-Batal et al. 2011)
*F. solani* (Mart*.) f.*sp. *phaseoli* (Burkholder)	Reduce pathogenicity, the ability of the Biological Control of Fusarium Root Rot	*Fusarium* sp.	0, 0.06, 0.09, 0.12, 0.15 and 0.18	(Ahari Mostafavi et al. 2012)
*Penicillium chrysogenum* NRRL 792	Improvement of alkaline protease production	Mainly used in detergents to facilitate the release of proteinaceous stains	0.02, 0.04, 0.06, 0.08, 0.1, 0.12, 0.14, 0.16	(Afifi et al., 2014)
*Trichoderma reesei* (PTCC 5142)	Enhance cellulases enzyme activity	Industrial enzyme production	0.25	(Shahbazi et al. 2014)
*Trichoderma harzianum* 65 (Th65) isolate (WT)	Stronger growth inhibition and colonization rate, Effect on the production of extracellular compounds and mycoparasitism enzymes	Soilborne Fungal Pathogens	0.25	(Abbasi et al. 2016)
*Trichoderma* variants: TvM1-UV1, TvM9-UV1, TvM1-UV2, and TvM9-UV2	Improve functional peptides and antagonistic activity	*Alternaria solani, Fusarium oxysporium,* and *Rhizoctonia solani*	0.25, 0.5, 0.75, 1.0, 1.5, 2.0, 2.5, 3.0, 4.0, 4.5 and 5.0	(El-Bialy et al. 2019)
*Trichoderma koningii* (NAS–K1)	Increased endochitinase enzyme activity	*Macrophomina phaseolina*	0.25	(Goharzad et al. 2019)
*Trichoderma aureoviride* Tv59	Improvement in biocontrol capabilities	*Fusarium graminearum, Sclerotinia sclerotiorum and Rhizoctonia solani*	0.25	(Soufi et al. 2021b)
*Trichoderma aureoviride* Tv59	Effective control of charcoal rot of sesame	*Macrophomina phaseolina*	0.25	(Soufi et al. 2021a)
mutant *Trichoderma* formulation	Reduced disease, increased growth plant of infected soybean	*Macrophomina phaseolina* (Tassi)	not mention	(Orojnia et al. 2021)
*Beauveria bassiana* (Balsamo)	Decrease the total carbohydrate, followed by total lipids and proteins of the pathogen	*Earias insulana* (Boisd.)	0.4 and 0.7	(Kandil et al., 2022)

Gamma radiation has been found to induce the expression of new or enhanced virulence factors in biocontrol agents, which leads to improved control of target plant pathogens and pests without posing risks to public health. For instance, the BM −15 mutant strain of *Bacillus thuringiensis*, which was mutated by the induction of gamma radiation (under 120 Gy), produces 2.6 times more chitinase than the wild type (Gomaa, 2021). Gamma mutants, under 0.5–3.0 kGy, of *Bacillus* and *Streptomyces* have been found to increase their antagonistic activity against *Macrophomina phaseolina* and *Fusarium oxysporum* f. sp. *udum* in pulses. Comparison of the mutant bacteria with the wild type showed that the synthesis of lipopeptides and hydrolytic enzymes, as well as swarming and swimming, were increased (Manikandan et al., 2022). The gamma mutants (under 0.1- 3 kGy) of *Bacillus subtilis* UTB1, M419, and M464 have better antifungal properties against *Aspergillus flavus* than the wild type. Production of iturin-like lipopeptides and swarm motility were increased, allowing them to colonize surfaces and reduce aflatoxin to a greater extent (Afsharmanesh et al., 2014). Induced gamma irradiation also resulted in increased production of biosurfactants and biofilms in mutants of *B. subtilis* UTB1 (Afsharmanesh et al., 2013). Moreover, integrating irradiation and antagonist treatments may increase their efficiency. Combining *Pseudomonas fluorescens* with gamma irradiation (under 600 Gy) was found to be more successful in postharvest control against *Penicillium expansum* than either treatment alone (Mostafavi et al., 2011). Furthermore, enhancement of volatile production by gamma radiation, under 2 kGy, in *Lactobacillus plantarum* had a promising result in controlling sap-stain fungi in wood stores and infected trees (El-Fouly et al., 2011).

Although the effect of gamma radiation on various biological control fungi has been studied, most of the studies have involved *Trichoderma* species. These studies have shown that the fungi greatly improved their ability to control plant diseases. The antifungal metabolites of *Trichoderma harzianum, Trichoderma viride*, and *Trichoderma koningii* mutants (under 0.2 and 0.5 kGy of gamma radiation) were assayed by HPLC. They produced highly active exo-enzymes and had the highest isozyme band number and quantity of chitinase and beta-1,3 glucanase (Haggag and Mohamed, 2002). Moreover, the efficacy of *Trichoderma* against *Alternaria solani, F. oxysporium*, and *Rhizoctonia solani* was improved by the use of gamma rays, and the antagonistic activity of the second-generation variants was higher than that of the first generation (El-Bialy et al., 2019). Antifungal metabolites (hydrolytic enzymes, antibiotics, and total phenols) in the gamma radiation mutant (under 0.2 and 0.5 kGy) of *T. harzianum* against *F. oxysporum* were increased under salt stress conditions (Mohamed and Haggag, 2005). Combined use of mutant (at gamma radiation doses under 250 Gy) *Trichoderma* strains and *Piriformospora indica* increased the defence-related enzyme activity and the content of chlorophyll a, chlorophyll *b*, carotenoids, and total soluble protein of cucumber against *Fusarium* wilt (Lava and Babaeizad, 2021).

## 6 Conclusion

Gamma irradiation is an effective method for reducing pathogen damage, and it is a safe, sustainable, and environmentally friendly option due to the lack of residual effects (World Health Organization, 1988). It can also induce new or enhanced biotic properties in biocontrol agents and natural predators, leading to improved pest and disease control in crops, without increasing the virulence of pathogens that could pose a risk to public health. However, the use of gamma irradiation has its limitations and challenges, such as high cost and technical requirements (Jeong and Jeong, 2018). Furthermore, the effects of gamma rays on biological control agents may not always be predictable and can vary significantly depending on the species, dose, and environmental conditions. In some cases, gamma irradiation may lead to the selection of strains with altered biotic or ecological fitness, potentially impacting their role in integrated pest management strategies. To overcome these challenges, it is crucial to develop cost-effective and accessible solutions for utilizing gamma irradiation. For instance, developing training programs to educate farmers on proper and safe use of gamma irradiation can help minimize the risk of crop damage. Moreover, selecting the appropriate radiation dose and applying it to beneficial microorganisms can increase their efficacy in biocontrol of plant diseases. Finally, developing alternative methods for controlling plant diseases that are less expensive and more accessible to farmers in developing countries could also be explored.

## Authors’ contributions

M.R. wrote the original manuscript. A.G. contributed to the review and editing. S.S. edited the final version manuscript.

## Declaration of competing interest

The authors declare that they have no conflict of interest. The research reported here did not involve experimentation with human participants or animals.

## Data Availability

The data that has been used is confidential.
